# E-Cigarette Awareness, Perceptions and Use among Community-Recruited Smokers in Hong Kong

**DOI:** 10.1371/journal.pone.0141683

**Published:** 2015-10-26

**Authors:** Man Ping Wang, William Ho Cheung Li, Nan Jiang, Lai Yan Chu, Antonio Kwong, Vienna Lai, Tai Hing Lam

**Affiliations:** 1 School of Nursing, University of Hong Kong, Hong Kong, Hong Kong; 2 School of Public Health, University of Hong Kong, Hong Kong, Hong Kong; 3 Hong Kong Council on Smoking and Health, Hong Kong, Hong Kong; Mario Negri Institute for Pharmacology Research, ITALY

## Abstract

**Background:**

Electronic cigarettes (e-cigarettes) are being increasingly used. We examined the correlates associated with e-cigarette awareness, use and perceived effectiveness in smoking cessation among Chinese daily smokers in Hong Kong.

**Methods:**

Daily smokers (N = 1,307) were recruited to a community-based randomised controlled trial (‘Quit to Win’) in 2014. Socio-demographic characteristics, conventional cigarette smoking status, nicotine addiction level, quit attempts, quit intention, e-cigarette awareness, use and perceived effectiveness on quitting were reported at baseline and 1-week follow-up. Multivariate logistic regression was used to identify factors associated with e-cigarette awareness, use and perceived effectiveness in quitting.

**Results:**

Most smokers (82.6%, 95% CI 80.2%-84.9%) had heard about e-cigarettes, and 13.3% (11.3%-15.5%) ever used e-cigarettes. Most users (74.1%) and non-users (91.2%) did not perceive e-cigarettes as effective in quitting. Being younger and having a larger family income were associated with e-cigarette awareness. Being younger, a tertiary education and a stronger addiction to nicotine were associated with e-cigarette use, which was itself associated with lower levels of intention to quit and had no association with attempts to quit (P for trend 0.45). E-cigarette use, the last quit attempt being a month earlier, having made a quit attempt lasting 24 hours or longer and perceiving quitting as important were all associated with the perceived effectiveness of e-cigarettes in quitting (all P <0.05).

**Conclusions:**

Among community-recruited smokers who intended to quit, awareness of e-cigarettes was high, but most did not perceive e-cigarettes as effective in quitting. Correlates concerning e-cigarette perceptions and use will help to inform prospective studies, public education and policy on controlling e-cigarettes.

## Introduction

Awareness of electronic cigarettes (e-cigarettes) was particularly prevalent among smokers (more than 90%) and was associated with being younger and better educated [1,2]. Although evidence of the long-term effects of e-cigarettes on health and smoking cessation was inconclusive [3], their use has been increasing in recent years. Short-term adverse effects of e-cigarette use were reported, particularly on cardiovascular and respiratory systems [4]. Current smokers are more likely to report e-cigarette use [5,6], and the dual use of conventional and e-cigarettes is of concern. Among current smokers, being female and younger and having a higher education level were associated with e-cigarette use [7–9]. However, associations with quit attempts and intention to quit are less consistent in the literature [8,9]. Qualitative studies have found that positive perceptions of e-cigarettes as cool/stylish, beneficial to smoking reduction or cessation, and less harmful than conventional cigarettes are the main reasons for their use among adults [10,11]. The perceived effectiveness of e-cigarettes in quitting may also affect their usage. Most published studies have focused on Western populations, with very few investigating e-cigarette use and perceptions on smoking cessation among Chinese smokers [12].

E-cigarettes were first invented in China, which has become the main producer [13]. The first manufacturer (the Ruyan Group) was established in Hong Kong, the most developed and Westernised city in China, with the lowest smoking prevalence (10.7% daily smokers in 2012) of developed countries due to a smoke-free policy, high tobacco tax and free smoking cessation services [14]. Smoking has been thoroughly ‘denormalised’ in Hong Kong, and smokers are becoming more addicted to nicotine [15]. E-cigarette use in smoke-free venues is prohibited under smoke-free legislation, although the law does not specify e-cigarettes as‘smoking’. Local social media websites promoting e-cigarettes are well received (e.g. more than 100,000 ‘likes’ for Hong Kong e-cigarettes on Facebook) [16], and the number of local anecdotal reports on e-cigarettes has grown dramatically from 35 in 2010 to 116 in 2014 (WiseNews search). E-cigarettes with nicotine should be registered as a drug before being sold, but there is no such registration yet. However, the loophole of purchase through the Internet is convenient and popular, with nicotine-free e-cigarettes also available in many shopping mall kiosks.

The present study aimed to investigate awareness of e-cigarettes, their level of use and their perceived effectiveness in quitting among community-recruited daily smokers in Hong Kong. We also examine the factors associated with such awareness, use and perceived effectiveness.

## Methods

### Ethical statement

Ethical approval was granted by the Institutional Review Board (IRB) of the University of Hong Kong and the Hospital Authority, Hong Kong West Cluster. Written informed consent was obtained from all respondents, with the procedure approved by the IRB (UW-14-382).

### Sampling

The ‘Quit to Win Contest’ is a popular worldwide project for promoting smoking cessation in the community [17]. We used data from the local Contest, a community-based project aiming to encourage smokers to quit through incentives and interventions, with community support. Recruitment to the project was conducted from March to September 2014 in all 18 Hong Kong districts. Adult Chinese daily smokers were proactively recruited in community settings by trained research staff. Smoking status was verified by exhaled carbon monoxide with 4 ppm or above indicating smoking. Of the 7572 smokers approached, 1315 (17.4%) were willing to participate and 1307 were eligible (i.e. aged 18 or older). Smokers completed questionnaires at baseline and received follow-up telephone interviews after a week to monitor smoking status. All data were obtained from the baseline survey except questions related to e-cigarette awareness, ever use and perceived effectiveness in quitting, which were measured at the one-week follow-up interview because of limited time at the baseline survey. Compared with daily smokers recruited by the government using probability sampling, our own sample showed a similar distribution (all Cohen’s effect sizes < 0.03) in the case of sex (female 19% vs 15%), age (mean 42.5 vs 46.3) and daily cigarette consumption (mean 15.7 vs 13.0) [18].

### Measurement

As the first few surveys on e-cigarette use among smokers, 3 simple questions about e-cigarettes were put. Smokers were asked if they had ever heard of such things by means of a description: ‘something like a real cigarette with smoke produced but without combustion’. This is similar to questions adopted by other studies assessing awareness [1,19]. Responses were divided into being aware (‘yes’) or not (‘no’ or ‘don’t know’). No levels of e-cigarette use were specified and smokers were expected to report any use at all, including even a single puff [20]. Those who answered ‘yes’ were defined as ‘ever users’. Smokers also reported whether they thought e-cigarettes could help them to quit smoking, again with responses of ‘yes’, ‘no’ or ‘don’t know’.

Details of cigarette smoking behaviour were collected, including duration of cigarette use, number consumed per day and nicotine addiction, as measured by the Heaviness Smoking Index (HSI) [21]. Times of the last quit attempt were recorded as ‘never, in the past month, one month ago, during the past year, a year ago’. We also investigated whether smokers had ever abstained for more than 24 hours as an indicator of a serious quit attempt. Intention to quit or reduce smoking in the past was categorised as ‘<7days, 8-<30 days, 31-<60 days, 60+ days, never’. Quitting self-efficacy was assessed by means of3 questions with scores of 0–10,higher scores indicating better self-efficacy in the case of questions about the perceived importance of quitting and confidence in successful quitting, while the reverse was true in the case of perceived difficulty of quitting. Methods used to quit smoking were assessed, including cessation clinics, telephone hotlines, nicotine replacement therapy, cessation medication, other cessation programmes—or none.

### Statistical analysis

STATA 13.0 was used for data analysis. Descriptive prevalence was weighted for the sex and age of general smokers from the government’s population 2012 survey. Confidence intervals (95% CI) for prevalence of e-cigarettes awareness, ever use and perceived effectiveness in quitting were estimated. Multivariate logistic regression was used to yield odds ratios (ORs) for e-cigarette awareness, use and perceived effectiveness. The factors examined included socio-demographic characteristics (e.g. sex, age, educational attainment and family income), and smoking and quitting behaviour. In adjusted models, sex, age, education and family income were mutually adjusted.

## Results

Among the 1307 daily smokers, the mean age was 46.0 (±14.0), most were male (83.7%), with a secondary education or above (81.3%) and a family income of HK$ 10,000–39,999 (66.4%) (US$ 1 = HK$ 7.8), consumed on average 16.4 (±9.8) cigarettes daily and had moderate levels of nicotine addiction (average HSI 2.8 ±1.6) (Table 1). Most had tried to quit (74.2%) and intended to quit or reduce smoking within 60 days (91.7%), but only 19.5% had ever used any cessation services. The prevalence of e-cigarette awareness was high (82.6%, 95% CI 80.2%-84.9%); 13.3% (95% CI 11.3%-15.5%) had ever used e-cigarettes and 11.0% (95% CI 9.2%-13.1%) perceived e-cigarettes as effective in quitting. Most smokers (88.0%), including e-cigarette users (74.1%) and non-users (91.2%), did not perceive e-cigarettes as effective in quitting.

**Table 1:** Socio-demographic characteristics, smoking, quit and e-cigarette use.

*Socio-demographic characteristics*		n	% ^a^	
			Un-weighted	Weighted
Male	-	1061	81.2	83.7
Age (mean)	-	1300	42.5(±15.2)	46.0 (±14.0)
Education	≤ Primary	198	15.5	18.7
	Secondary	822	64.2	64.0
	≥ Tertiary	261	20.4	17.3
Family income (HK $)	<10000	242	20.0	21.0
	10000–19999	428	35.3	36.1
	20000–39999	378	31.2	30.3
	≥40000	163	13.5	12.7
***Smoking***				
Duration of smoking (mean year)	-	1288	25.0 (±15.0)	28.2(±14.2)
Daily cigarette consumption (mean)	-	1306	15.7 (±9.4)	16.4 (±9.8)
Morning smoking	<5 minutes	576	44.7	46.7
	6–30 minutes	313	24.3	23.9
	31–60 minutes	146	11.3	11.5
	>1 hour	254	19.7	17.9
Heaviness smoking index (mean)		1288	2.7 (±1.6)	2.8 (±1.6)
Heard about e-cigarettes	-	856	83.5	82.6
Ever use e-cigarettes	-	154	15.0	13.3
Perceived e-cigarettes as effective on quitting	Yes	119	11.6	11.0
	Don’t know	432	42.3	43.6
	No	471	46.1	45.4
***Quitting characteristics***				
Last quit attempt	<1 month	113	8.7	8.5
	<1 year	218	16.7	16.2
	≥1 year	638	49.0	50.0
	Never	333	25.6	25.2
Quitting for more than 24 hours	-	954	73.0	73.4
Intention to quit / reduce smoking	<7 days	875	67.3	68.1
	<30 days	238	18.3	18.1
	<60 days	75	5.8	5.5
	60+ days or never	113	8.7	8.3
Perceived importance of quit (0–10)	-	1303	7.9 (±2.2)	7.9 (±2.2)
Confidence of successful quit (0–10)	-	1301	5.8 (±2.4)	5.8 (±2.4)
Perceived difficulty of quit (0–10)	-	1301	7.3 (±2.5)	7.3 (±2.5)
Assisted quit methods (multiple choices)	Cessation clinics	27	2.8	3.1
	Telephone hotlines	25	2.6	2.4
	Nicotine replacement therapy	117	11.9	12.6
	Cessation medication	34	3.5	3.5
	Other cessation program	6	0.6	0.5
	Any of above	183	18.7	19.5

^a^ All are % except otherwise indicated.

Younger age and higher family income were associated with increased adjusted odds of e-cigarette awareness (all P<0.01) (Table 2). Being younger and having a higher level of education were associated with increased odds of e-cigarette use (P for trend <0.01). None of the socio-demographic factors were associated with perceived effectiveness. Nicotine addiction was associated with awareness of e-cigarettes (AOR 1.16, 95% CI 1.03–1.30) and ‘ever use’ (AOR 1.30, 95% CI 1.14–1.48) (Table 3). The duration of smoking was marginally associated with awareness (AOR 1.04, 95% CI 1.01–1.07). Making the last quit attempt < 1 year and 1+ years were associated with AORs (95% CI) of 2.58 (1.28–5.18) and 2.24 (1.22–4.11) for perceived effectiveness of e-cigarette in quitting, respectively. Making a quit attempt lasting for 24 hours was also associated with AOR of 2.11 (1.20–3.68) in perceived effectiveness. As expected, e-cigarette ever use was strongly associated with perceived effectiveness in quitting (AOR 3.24, 95% CI 2.03–5.17). The use of cessation services and self-efficacy in quitting were not associated with e-cigarette awareness, use or perceived effectiveness, except for a marginal association of perceived importance with perceived effectiveness in quitting. E-cigarette use was negatively associated with smoking intention (AOR 2.16 95% 1.05–4.44 in having the intention to quit for 30+ to < 60 days), and not associated with attempting to quit.

**Table 2:** Socio-demographic factors associated with e-cigarette awareness, use and perceived effectiveness on quitting.

	Awareness		Use		Perceived effectiveness	
	OR (95% CI)	AOR (95% CI) ^a^	OR (95% CI)	AOR (95% CI) ^a^	OR (95% CI)	AOR (95% CI) ^a^
Sex						
Male	1	1	1	1	1	1
Female	1.24 (0.79–1.96)	0.95 (0.59–1.55)	1.43 (0.94–2.16)	1.06 (0.67–1.68)	1.48 (0.94–2.35)	1.18 (0.71–1.98)
Age	0.96 (0.95–0.97)***	0.97 (0.95–0.98)***	0.97 (0.96–0.98)***	0.97 (0.96–0.99)**	0.99 (0.97–1.00)	0.99 (0.98–1.10)
Education						
≤ Primary	1	1	1	1	1	1
Secondary	2.02 (1.34–3.04)**	1.05 (0.64–1.72)	1.96 (1.04–3.67)*	1.58 (0.72–3.44)	1.32 (0.71–2.45)	1.35 (0.63–2.89)
≥Tertiary	3.38 (1.89–6.04)***	1.11 (0.54–2.28)	3.86 (1.97–7.58)***	2.78 (1.17–6.60)*	2.04 (1.03–4.04)*	1.83 (0.76–4.44)
P for trend	<0.001	0.77	<0.001	<0.01	0.03	0.15
Family income (HK $)						
<10000	1	1	1	1	1	1
10000–19999	1.46 (0.95–2.26)	1.01 (0.63–1.62)	1.14 (0.67–1.95)	0.83 (0.47–1.46)	1.52 (0.82–2.83)	1.36 (0.71–2.59)
20000–39999	2.98 (1.82–4.88)***	2.05 (1.21–3.49)**	1.19 (0.70–2.04)	0.76 (0.44–1.38)	1.10 (0.57–2.12)	0.94 (0.47–1.86)
≥40000	3.54 (1.75–7.17)***	2.07 (0.95–4.50)	1.57 (0.83–2.94)	0.76 (0.38–1.53)	2.21 (1.09–4.48)*	1.65 (0.75–3.59)
P for trend	<0.001	<0.01	0.19	0.41	0.15	0.62

^a^ Adjusting for sex, age, education and income.

* P<0.05,

** P<0.01,

*** P<0.001.

**Table 3:** Smoking and quitting factors associated with e-cigarette awareness, use and perceived effectiveness on quitting.

	Awareness		Use		Perceived effectiveness	
	OR (95% CI)	AOR (95% CI) ^a^	OR (95% CI)	AOR (95% CI) ^a^	OR (95% CI)	AOR (95% CI) ^a^
Duration of smoking	0.97 (0.96–0.98)***	1.04 (1.01–1.07)**	0.97 (0.96–0.99)***	1.10 (0.69–1.74)	0.98 (0.97–1.00)	0.97 (0.94–1.00)
Heaviness smoking index	1.11 (1.00–1.23)*	1.16 (1.03–1.30)*	1.15 (1.03–1.29)*	1.30 (1.14–1.48)***	1.08 (0.96–1.22)	1.11 (0.97–1.27)
Last quit attempt						
Never	1	1	1	1	1	1
≥1 year	1.48 (1.00–2.18)	1.43 (0.94–2.18)	1.36 (0.87–2.12)	1.38 (0.86–2.23)	1.96 (1.14–3.38)*	2.24 (1.22–4.11)*
<1 year	1.61 (0.95–2.73)	1.47 (0.83–2.63)	0.89 (0.48–1.63)	0.76 (0.39–1.45)	2.24 (1.18–4.25)*	2.58 (1.28–5.18)*
<1 month	1.28 (0.69–2.39)	1.20 (0.60–2.37)	2.16 (1.18–3.96)*	1.80 (0.94–3.46)	1.40 (0.60–3.23)	1.03 (0.52–3.27)
P for trend	0.18	0.33	0.11	0.45	0.15	0.22
Ever quit for ≥24 hours						
No	1	1	1	1	1	1
Yes	1.41 (0.98–2.02)	1.35 (0.91–1.98)	1.37 (0.90–2.06)	1.31 (0.84–2.04)	1.88 (1.14–3.11)*	2.11 (1.20–3.68)*
Intention to quit/reduce						
<7 days	1	1	1	1	1	1
<30 days	0.90 (0.59–1.39)	0.77 (0.48–1.22)	1.05 (0.66–1.67)	0.92 (0.55–1.55)	1.30 (0.80–2.10)	1.22 (0.72–2.06)
<60 days	3.03 (0.93–9.93)	2.56 (0.76–8.59)	1.94 (0.98–3.86)	2.16 (1.05–4.44)*	1.56 (0.70–3.45)	1.41 (0.60–3.31)
No	0.87 (0.50–1.51)	0.73 (0.40–1.33)	1.40 (0.81–2.45)	1.48 (0.83–2.64)	0.72 (0.34–1.54)	0.66 (0.29–1.50)
P for trend	0.90	0.56	0.09	0.08	0.93	0.69
Assisted quit methods						
None	1	1	1	1	1	1
Any	0.94 (0.57–1.54)	1.01 (0.59–1.72)	1.37 (0.87–2.18)	1.38 (0.83–2.28)	1.34 (0.81–2.23)	1.46 (0.86–2.50)
Perception on quitting						
Importance	1.05 (0.98–1.13)	1.06 (0.99–1.15)	1.06 (0.98–1.15)	1.08 (0.38–1.57)	1.14 (1.03–1.26)*	1.16 (1.04–1.29)*
Confidence	1.01 (0.94–1.08)	1.04 (0.96–1.12)	0.94 (0.88–1.01)	0.95 (0.88–1.02)	1.01 (0.93–1.09)	0.99 (0.91–1.08)
Difficulty	1.05 (0.98–1.12)	1.02 (0.96–1.10)	1.00 (0.94–1.08)	1.00 (0.92–1.07)	1.02 (0.95–1.10)	1.02 (0.94–1.11)
E-cigarette use						
No	-	-	-	-	1	1
Yes	-	-	-	-	3.44 (2.23–5.29)***	3.24 (2.03–5.17)***

^a^ Adjusting for sex, age, education and income.

* P<0.05,

** P<0.01,

*** P<0.001.

## Discussion

This Chinese sample of community-recruited daily smokers shared similar demographic and smoking characteristics with smokers in the general Hong Kong population. The prevalence of e-cigarette awareness (82.6%) was comparable with that observed among smokers in the United States (US) (91.3%), the United Kingdom (UK) (90.5%) and Italy (96.0%), but was higher than among Australian smokers (64.8%) [6,22,23]. Such high prevalence of awareness was unexpected, given that e-cigarette promotion was not widespread and no large-scale mass media promotion was observed in Hong Kong. The dissemination of information among smokers may be fast because of Hong Kong’s small geographical area and the popular use of mobile communication devices to share information [24]. Younger age and higher family income were associated with awareness, and these two factors also correlated with communication technology use [24]. Studies also suggested the e-cigarette industry was targeting young people and increasingly investing in advertising their products [25]. We did not record sources of information about e-cigarettes because of the brief questionnaire design. Other studies have suggested information on e-cigarettes is spread widely through social media [26], which is consistent with our observation of the popularity of local e-cigarette Facebook postings [16]. Further studies are warranted to investigate the content of such shared information and its impact on e-cigarette use.

The prevalence of e-cigarette ever use is much lower among smokers in Hong Kong (13.3%) compared with that in the US (36.5%), UK (43.3%), Australia (34.7%) and Italy (20.4%) [6,22,23]. This is probably due to restrictions on the nicotine content in Hong Kong while other countries (e.g. the US or UK) have looser regulations. Stringent legislation banning smoking, interpreted by the government to include e-cigarettes in smoke-free areas in Hong Kong, may also contribute to the low prevalence of e-cigarette use. Young and better educated smokers were more likely to use e-cigarettes, which is consistent with other studies [7–9]. These groups of smokers may have better health awareness and are thus more likely to use e-cigarettes as a means of reducing the harm from smoking, which is a hallmark of e-cigarette advertising [27] used to target young users [25].

Unlike Western studies showing male smokers to be more susceptible to e-cigarettes [5], we found the same levels of e-cigarette use among female and male smokers. Given that female smoking prevalence is very low (3.1%) and highly denormalised in Chinese culture [28], e-cigarettes probably act as a substitute for conventional cigarettes, leading to higher than expected use in females. The positive association between nicotine addiction and e-cigarette use suggests heavy smokers are more likely to consider e-cigarettes as an aid to cessation, because of marketing promotion of its cessation function, although the evidence for this is inconclusive. The notion of reverse causation, whereby e-cigarette use perpetuates nicotine addiction, is possible. This is consistent with the negative association between e-cigarette use and intention to quit, and the lack of association with quit attempts suggests e-cigarette use may not be associated with quitting behaviour. However, further studies are needed to confirm these observed associations.

Compared with awareness of e-cigarettes, perceived effectiveness in quitting was much less prevalent. This suggests e-cigarettes are not regarded as an effective tool for cessation by most smokers, among both e-cigarette users and non-users in Hong Kong. As expected, e-cigarette use was strongly associated with perceived effectiveness in quitting. It is uncertain whether the association reflects experience of usage or is due to smokers perceiving e-cigarettes as an effective means of quitting and thus being more likely to try them. Similarly, temporal associations of perceived effectiveness with quit attempts and perceived importance of quitting were uncertain. Longitudinal studies are warranted to clarify the associations of e-cigarette use and quit attempts with perceived effectiveness in quitting. Nevertheless, our study is one of the few from non-Western populations to provide important findings on e-cigarette use.

The study has several limitations. First, non-random sampling methods were used to recruit daily smokers, although their sample characteristics were similar to those of smokers in the general Hong Kong population. Smokers are increasingly difficult to reach in the territory because of the low smoking prevalence. Our study, with a moderately sized sample of smokers, provided valuable information on e-cigarettes in a group of smokers motivated to quit. The prevalence of awareness and e-cigarette use in this study may not be directly comparable with studies using representative sampling methods [6,22,23]. Second, all information was self-reported. Although questions on conventional smoking behaviour including intention to quit, quit attempts and nicotine addiction, had been validated in other studies, the questions on e-cigarettes had not been validated. Third, the temporal sequence of correlates associated with e-cigarette awareness, use and perceived effectiveness are not clear in this cross-sectional survey.

## Conclusions

Among community-recruited smokers intending to quit, awareness of e-cigarettes was high and some had used them for the purpose, but most did not perceive them as an effective aid to quitting. E-cigarette use was negatively associated with quit intentions and not associated with quit attempts. Although still needing to be confirmed by further research, the study provides some findings on correlates (e.g. young age, better education and higher income) concerned with e-cigarette awareness and use which may help in designing prospective studies, public health education and public policy on controlling e-cigarettes.
